# Identifying best practices for substance-related intimate partner violence screening and referral: a narrative review

**DOI:** 10.3389/fpsyt.2024.1380102

**Published:** 2024-06-18

**Authors:** Amber M. Jarnecke, Tanya C. Saraiya

**Affiliations:** ^1^ Department of Psychiatry & Behavioral Sciences, Medical University of South Carolina, Charleston, SC, United States; ^2^ Center of Alcohol & Substance Use Studies, Rutgers University-New Brunswick, Piscataway, NJ, United States

**Keywords:** intimate partner violence, substance use, substance use disorder, screening, best practices

## Abstract

**Introduction:**

Substance use is strongly associated with intimate partner violence (IPV) and is a modifiable risk factor for IPV. However, lack of comprehensive screening and referral for co-occurring IPV and substance use, along with their psychiatric sequalae, limits the identification and implementation of effective interventions for substance-related IPV. This narrative review (1) investigates the literature on screening and referral practices for IPV, and if these include screening for substance use or other psychiatric comorbidities, (2) provides recommendations for current best practices, and (3) suggests future directions for research and practice aimed at identifying and reducing substance-related IPV.

**Methods:**

A narrative literature review examined studies investigating IPV screening and referral programs in clinics. Selected studies were reviewed for: (1) effectiveness, (2) barriers to implementation and sustainability, and (3) responsivity to psychiatric comorbidity, including substance use and substance use disorders (SUD).

**Results:**

Findings suggest that effective IPV screening and referral programs have been developed, but disparities in IPV screening exist and many programs only screen for IPV victimization. Barriers to the implementation and sustainability of IPV screening programs include lack of ongoing provider training, funding or institutional support, and direct connection to referral services. Further, many IPV screening programs lack assessment of and referral for comorbid psychiatric conditions, including substance use, and tend not to be routinely implemented in SUD clinics.

**Discussion:**

Additional systematic work is needed to develop universal and comprehensive screening and referral programs for substance-related IPV and address issues of long-term sustainability, particularly within SUD treatment settings.

## Identifying best practices for substance-related intimate partner violence screening and referral: a narrative review

Intimate partner violence (IPV) affects over 12 million individuals annually and is associated with substantial individual, family, community, and public health costs (1). Consequences of IPV can include chronic nervous system, reproductive, cardiovascular, and chronic pain conditions as well as posttraumatic stress disorder (PTSD), depression, suicidality, anxiety, and substance use (2–5). These health costs are compounded by the social costs of IPV: housing instability, relationship conflict, financial dependence or insecurity which can prevent partners from leaving IPV situations, loss of child custody, and unemployment (6). IPV has a direct effect on parenting and childhood development and can contribute to an intergenerational transmission of trauma (7). Men and women tend to report similar rates of psychological and physical IPV, but women tend to experience more physical consequences of IPV victimization and report higher rates of sexual IPV victimization than men (8). Individuals with marginalized or stigmatized identities tend to report higher rates of IPV, and risk of IPV is highest among adolescents and young adults who hold multiple marginalized or stigmatized identities (9). Black, Indigenous, and other people of color (BIPOC) who experience IPV are at a substantially increased risk of not only dying due to IPV, but also dying earlier than their White counterparts (10). Together, IPV and its psychological, physical health, and structural consequences costs the U.S. $8 billion annually in direct healthcare expenditures and lost productivity (11, 12). Moreover, rates of IPV have only increased since the COVID-19 pandemic, suggesting a serious and growing need to explore ways to identify, prevent, and treat IPV, particularly amongst the most vulnerable populations (13, 14).

It is well established that substance use is commonly associated with IPV. Individuals who survive IPV may use substances to self-medicate the consequences of IPV (e.g., pain, depressive symptoms, trauma symptoms, etc.) (15–19). Recent research in a large sample of women (N = 13,597) demonstrated that IPV during the COVID-19 pandemic was associated with a 17% increase in using substances to cope with stress (20). It is also well established that IPV exposure often occurs in the context of substance use, and thus substance use is a modifiable risk factor for IPV (21, 22). Treating substance use disorders (SUD) or integrating content about IPV into SUD treatment reduces IPV perpetration, whereas active IPV interferes with treatment engagement and retention among individuals with SUD (16, 19, 23). Given extensive data demonstrating strong, bidirectional links between substance use and IPV, there is a need for comprehensive screening and referral programs for co-occurring IPV and substance use (19, 24–28).

The current narrative review examines extant screening and referral practices for IPV and examines if such programs are routinely implemented into SUD treatment settings or incorporate screening for substance use or other co-occurring psychiatric conditions. We provide recommendations for current best practices and detail future directions for identifying and reducing substance-related IPV. We focus primarily on studies investigating IPV screening and referral programs utilized in real-world clinical settings (compared to screening programs that were only used as part of a research protocol). Selected studies highlight: (1) effectiveness of existing IPV screening programs, (2) barriers to implementation and sustainability of IPV screening programs, and (3) responsivity (or lack thereof) of IPV screening programs to co-occurring psychiatric conditions, including substance use and SUD.

## Current screening and referral practices

### Effectiveness

In general, when IPV screening protocols are implemented in clinical settings, they tend to reach patients (i.e., patients are screened). A recent systematic review of IPV screening among women in clinical settings found that a median of 80% of all eligible women were screened across settings and the median percentage of women who disclosed IPV victimization was 11% (29). However, rates of screening may differ by race or ethnicity and be biased toward screening White women over Black or Latina women and English-speaking women over non-English speaking women (30–32). When comprehensive screening programs are utilized (i.e., screening programs that have institutional support, use validated screening measures, offer continued provider training, enable immediate connection with referral support services), IPV screening and disclosure rates increase, as do providers’ ratings of their self-efficacy in performing IPV screenings (33). There is some mixed evidence on the effectiveness of IPV screening programs on patient referral uptake. This literature is clouded by different operational definitions of ‘successful referral uptake’ (34–36). For instance, one systematic review found that among women who disclosed IPV, the median rate of referral to follow-up psychosocial services is 32%; and among women referred, 54% attend or receive psychosocial services (29). However, this investigation found fewer women are provided referrals or follow-up on referrals than the total number of women who disclosed IPV (29). Thus, there is room for improvement in screening practices, referral, and referral uptake services.

Most studies have examined the effectiveness of IPV victimization screening and fewer studies have systematically investigated the effectiveness of IPV perpetration screening programs (37). When screening for IPV perpetration does occur, it tends to bias screening men (38, 39). However, some healthcare systems, such as the Veterans Health Administration, are conducting IPV perpetration screening across gender (40). In contrast, IPV victimization screening tends to bias screening women, and little research has examined IPV victimization screening programs in real-world clinical settings that are inclusive of cisgender men, nonbinary, transgender, and gender diverse individuals. Among a survey of men in England, only 1.6% of men reported they had ever been asked about IPV from a healthcare provider (41). One study examined the effectiveness of a universal IPV assessment in a pediatric acute care setting where all child caregivers (N = 169,333 visits) were assessed on two items related to violence in the home (42). Of true-positive screens, 91% of individuals who disclosed violence in the home were female caregivers and the remaining 9% of individuals who disclosed violence were male caregivers (42). This suggests that while women have been traditionally the subject of IPV victimization screening practices, men also disclose IPV victimization when screened.

Indeed, another set of investigations on universal IPV screening in Level I trauma centers across the U.S. found that women were more likely to screen positive for IPV and sexual violence victimization than men, but there was no gender difference between men and women who were physically hurt related to violence (43). Moreover, in a study that took place across a two-year period in emergency departments (N = 10,744), it was found that 6.7% of men reported IPV victimization or aggression and 8.7% of women reported IPV victimization or aggression (44). These gender differences in disclosure rates may reflect underreporting among men due to stigma, fear of consequences, desire for privacy etc. (45). However, research examining attitudes related to help seeking among men in England (N = 1,368) suggest that a substantial proportion of men think that healthcare providers should ask all patients about IPV (27%) or should ask all patients if they present symptoms consistent with IPV exposure (65%) (41). Findings across these studies show that men not only experience and disclose IPV victimization, but also show some similar health consequences to IPV as women.

Among transgender, nonbinary, and gender diverse individuals, research shows significant disparities in rates of IPV, such that transgender individuals are twice as likely to experience IPV relative to cisgender individuals (46). Risk of IPV further increases among LGBT+ individuals who hold other marginalized identities (9). Most data on the prevalence of IPV experiences among nonbinary, transgender, and gender diverse individuals has primarily been limited to survey-based research methods, but one study examining the effectiveness of IPV victimization screening and referral among transgender and gender diverse patients (N = 1,947) in a primary care setting identified that nearly 12% of patients screened positive for IPV, and among those patients 48.5% received a referral to psychosocial services and 63% who received an internal referral engaged in services (47). This study additionally identified that transgender and gender diverse patients assigned female at birth were less likely to receive a referral than patients assigned male at birth, and secondly that non-binary patients were more likely to receive a referral compared to binary patients. This suggests that provider practices and bias may be driving disparities in IPV screening even within transgender and gender diverse communities. It is important to note that IPV disparities among transgender and gender diverse individuals may be associated with increased discrimination, stigma, and rejection and are not related to anything inherent to the individual (46, 48, 49). This discrimination extends to social services centers, with research demonstrating that transgender and gender diverse individuals are more likely to experience unequal treatment in healthcare settings, domestic violence programs, and rape crisis centers (50, 51). Given increased risk for IPV among nonbinary, transgender, and gender diverse populations there is an urgent need for IPV screening programs to be gender-affirming and unbiased toward an individual’s gender.

While IPV screening and referral programs, when implemented consistently, are generally found to be effective, additional research suggests that IPV screening and referral practices may not be routinely implemented and studied in SUD clinics. A recent review of IPV screening programs across real-world healthcare settings did not identify any studies that included frontline providers conducting IPV assessments with patients in SUD treatment settings (29). Supporting the need for IPV screening programs among SUD patient populations, data show that individuals with a SUD in an emergency department are among the least likely of all patient presentations to be appropriately screened for IPV (52).

Despite the dearth of literature assessing IPV screening programs in SUD treatment settings, some studies have identified suitable assessments that could be adopted into substance use treatment clinics. For instance, one study validated the use of a 4-item IPV screener (i.e., the Jellinek Inventory for Assessing Partner Violence; J-IPV) across two substance use treatment settings and found that the measure demonstrated good psychometric properties to detect both IPV victimization and perpetration (53). Another study examined the comparative efficacy of a single session computerized IPV Screening, Brief Intervention, and Referral to Treatment (SBIRT) compared to one delivered by a case manager among women using substances in probation and community court sites (N=191) (54). Findings showed that both delivery methods were comparable and showed high IPV victimization detection rates (77%). Moreover, there was a significant association between screening and increased receipt of IPV services, social support, and abstinence from substances (54). While promising, the limitations of this study were its focus on solely women and lack of integration into standard SUD treatment settings (29, 54). Additional research is needed to assess the effectiveness of IPV screening and referral programs in real-world SUD clinics.

### Barriers to implementation

There are several barriers related to the successful implementation and sustainability of IPV screening programs in SUD treatment and other healthcare settings. Most of the research to date on implementation barriers to IPV screening has been conducted in settings such as primary care clinics, obstetrics and gynecologic clinics, and emergency departments, and fewer studies have examined barriers specific to SUD treatment settings. One systematic review identified that barriers to IPV screening included: provider’s personal barriers (e.g., provider discomfort in talking about IPV with patients), resource barriers (e.g., lack of time, training, or referral/follow-up resources to offer patients), attitudes and perceptions (e.g., belief that it is not providers’ role to screen, that there are more pressing issues to address, or that IPV is rare or patients do not want referrals for IPV-related concerns), fears (e.g., fear of not maintaining patient’s privacy, fear of police or social services involvement if IPV needs to be reported), and patient-related barriers (e.g., language barriers, patients would not report IPV, patients would stay with perpetrator) (55). Another study examined both provider and patient barriers and facilitators in implementing IPV perpetration screening (37). Providers identified the importance of training on IPV perpetration screenings, how these should be documented, and how to follow-up on positive screens. They identified that lack of time and seeing IPV as irrelevant to their role/patient presentation as significant barriers to screening. Patients noted the need for rapport with provider, a clear and comprehensive process and discussion of the consequences of screening/disclosure, and a preference for self-report screeners versus interview-only format.

Additional studies that have been conducted to examine barriers to implementing IPV screening have identified similar themes to those outlined above and further highlighted institutional barriers to IPV screening (e.g., administrator or leadership support for screening practices) and a need for culturally responsive assessment and care practices (37, 56–59). Telehealth-delivered services present their own challenges to the successful implementation of IPV screening and referral programs, particularly since increased utilization of telehealth since the COVID-19 pandemic. Telehealth makes it more difficult to ensure patient privacy, so some providers may be unable to complete an IPV screening if someone else is near the patient during their appointment (60). Others report significant concerns about telehealth since partners perpetrating IPV can track patients’ Internet activity (60).

Another considerable factor related to the success of IPV screening programs lies in their ability to provide direct and immediate connection with IPV-support services (e.g., shelters, mutual aid programs). Difficulties in linking clients from screening to referral uptake may contribute, in part, to why there are mixed findings with the effectiveness of IPV screening programs. In many geographic areas there are not enough IPV support centers, shelters, and resources for providers to refer patients (and even fewer that offer services for substance-using individuals, men, gender-diverse individuals, or are viewed as “safe spaces” for LGBT+ individuals) (61–66). Relatedly, providers may not have the time or resources to be knowledgeable about the full breadth of support services in their communities with which they can connect patients or may not have received training in how to adequately address patient reports of IPV and connect them with needed services (67–70). Further, many existing resource centers are capped in patient caseload and are unable to accept new referrals, thereby stagnating options for someone in active IPV situations.

Within SUD treatment settings, research has further identified that lack of provider training on the co-occurrence of IPV and SUD, including how intoxication increases risk of IPV perpetration, how substances/substance use may be used as part of coercion tactics, and how substances may be used to numb physical and/or emotional pain associated with IPV (71, 72), is a major barrier to implementing successful IPV screening programs. Additionally, relatively short treatment durations and lack of IPV service organizations that accept substance-using clients are barriers (66, 70). Increased stigma associated with the co-occurrence of IPV and SUD further represent a challenge in implementing and sustaining appropriate IPV screening and referral practices (66, 70, 72). Providers may be hesitant to ask questions about IPV experiences due to this stigma and patients may fear serious social or legal consequences should they report co-occurring IPV and SUD.

### Responsivity to co-occurring conditions

While substance use and SUD commonly co-occur with IPV, a number of other psychiatric conditions (e.g., depression, PTSD, suicidality) co-occur with IPV as well (46, 73–75). Data show that men and women in SUD treatment settings are more likely to report depression, anxiety, suicidality, and physical health problems when they have experienced IPV compared to individuals in these treatment settings who do not report IPV (76). Another study found that IPV was associated with an increased likelihood of seeking SUD treatment and of recent substance use in a sample of N = 452 transgender adults (77). While this research suggests there is a need to attend to both substance use and mental health concerns for IPV-exposed individuals, some providers may report only screening for substance use or IPV and few evidence-based IPV screening programs incorporate comprehensive screening for substance use or other mental health concerns (70, 78). This represents a critical area of future work, as the accurate identification of the mental health consequences of IPV is crucial to guide appropriate referrals and promote patient health.

## Recommendations for best practices and future directions

### Guidelines for screening

Current clinical practice guidelines by national and professional organizations (e.g., United States Preventative Services Taskforce; Veterans Affairs; American Medical Association) recommend health care providers screen for IPV in girls and women of reproductive age (79–82). Although stronger research evidence is needed to support recommendations for universal IPV screening, it is well established that IPV does not discriminate based on gender, race, ethnicity, sexuality, age, or any other factor, and that all individuals could be at risk of experiencing both IPV victimization and perpetration (1, 46, 83). Further, most studies demonstrate benefits to IPV screening and intervention with little risk of adverse events (34, 35), and most patients express a desire for IPV screening, indicating that screening facilitates their readiness for disclosure and engagement in IPV-related services (84).

Experts in the field have already done considerable work to document the need for universal IPV screening and to establish guidelines, or protocols, for screening and intervening IPV among diverse populations. For example, a study of N = 302 U.S. men who reported experiencing physical IPV victimization in a relationship with a female partner found that those who sought help for IPV from mental and medical health providers had more positive help-seeking experiences, whereas those who sought help from a domestic violence service system had the least positive help-seeking experiences (85). This research suggests when men disclose IPV and seek help from healthcare providers they tend to have positive experiences and that there may be a need for IPV-service agencies to offer some appropriate services and resources for men. Additionally, men in the study who reported more positive help-seeking experiences reported lower levels of alcohol use while those who had more negative experiences had higher PTSD symptoms (85). This points to the downstream effects of universal IPV screening practices on substance use and psychiatric sequelae. Nevertheless, although pilot guidelines for IPV screening among men have been proposed, they have not been routinely implemented (86).

Further demonstrating the need for universal IPV screening practices, other research surveying professionals in domestic violence prevention or intervention networks (N = 54) demonstrated that many professionals have little to no training on IPV among LGBT+ populations and feel their agencies or programs lack the appropriate resources, referrals and training to work with LGBT+ individuals experiencing IPV (63). Guidelines for IPV screening among LGBT+ individuals (87) and a transgender-specific IPV screening tool have been developed as well (88), but are likely underutilized in standard practice.

Finally, despite data suggesting that individuals with SUD may be among the most vulnerable clinical patient populations for experiencing IPV, they are also among the most least likely to be screened (16–19, 52). IPV screening may not be routinely implemented in SUD treatment settings (29, 78). There is a dearth of literature documenting best practices for integrating IPV screening programs into SUD clinics and, to our knowledge, no studies to date have specifically adapted IPV screening and referral programs to best meet the needs of patients with SUD. Thus, it has yet to be determined if existing IPV screening programs, utilized in other clinical settings, meet the needs of patients and providers in SUD clinics or if novel programs need to be developed.

Given the evidence pointing to the prevalence rates, need, desire, and effectiveness of IPV screening among all people, we first advocate in this article for universal IPV screening, regardless of one’s identity or presenting/clinical characteristics, and for future research to support this effort. Population-specific IPV guidelines, training, and screening tools, such as those referenced above, should be tested across larger samples and if found to be effective can be administered routinely, as appropriate. This includes using IPV screening tools in a patient’s primary language. Translation of validated IPV screening tools and development of new tools across language is critically needed (89). Developing standardized and evidence-based IPV screening and referral programs specifically for SUD treatment settings also represents a significant area of investigation for future research and clinical practice. Few IPV screening measures (see 90 for a review of existing, validated IPV assessments) have been validated in SUD populations, and it is necessary to determine how these measures perform in SUD patient populations. Finally, ensuring that IPV screening programs assess for IPV victimization as well as perpetration across SUD and other healthcare settings may enhance detection, inform appropriate referral and treatment, and reduce subsequent IPV and its consequences (37, 40, 91).

### Eliminating implementation barriers

Many barriers to the successful implementation of IPV screening and referral programs have been identified, but growing research highlights effective strategies for addressing these barriers. For instance, instilling institutional or clinic awareness of IPV and the co-occurrence of IPV with substance use and mental health conditions, continuous training for IPV screening procedures and protocols that are used following a positive IPV screen, and using culturally-responsive IPV assessment and validated screening tools paired with provider follow-up may address some barriers and are valued by both patients and providers (33, 37, 58, 92). For telehealth visits, environmental safety checks (e.g., asking if anyone else is in the room) can be used to assess privacy during telehealth appointments before IPV screening begins (93, 94), and technological screening and safety planning tools (e.g., safety plan smartphone applications) may increase access to IPV assessment and intervention whether patients present in person or via telehealth (30, 93, 95–97). Prior research shows that computerized IPV screening is non-inferior (or equivalent) to in-person IPV screenings by providers (54). One recent study of pregnant and postpartum women (N = 3,535) in a obstetrics and gynecology clinic within an academic medical center compared an in-person and text messaging-based SBIRT for mood, substance use, and IPV and found that the text-messaging service outperformed in-person screenings in the number of patients screened, number screened positive, and number referred to treatment (30). In addition, African American/Black women compared to White women in this sample were more likely to screen positive for IPV and receive referrals when they completed the text-messaging based SBIRT versus in-person screening from providers, suggesting the standardized text-messaging based may have helped reduced bias and racial disparities in screening. Thus, appropriate technological tools for IPV screening and referral could eliminate many barriers, and potential biases or inequities, for providers and patients in SUD clinics and other healthcare settings.

Additionally, given that a major barrier in effective IPV screening and referral is lack of immediate, direct connection with IPV-support agencies, more work is vital to fill this gap. Macy and Goodbourn (2012) conducted a literature review on strategies for promoting strong collaboration between substance use treatment and domestic violence service centers. The following strategies were identified to enhance partnership: training providers in both sectors on the co-occurrence and screening of IPV and substance use, interagency consultation and liaisons to facilitate collaboration and coordinated care, policies and funding to support training, interagency collaboration and resources/programs, and increased research on integrated care and the consequences of substance use recovery on IPV victimization (98). These findings may serve as a framework for future implementation science research to establish training programs and enhance linkage between SUD clinics and IPV-service agencies. Coordinated community responses (CCRs) (i.e., advocacy programs, law enforcement, other social service agencies coordinating responses to IPV or other forms of domestic violence) represent another option for increasing connection between clinics and IPV services. CCRs have been widely used, but research suggests that the evidence behind this model is limited by wide variability in its components and implementation and lack of theoretical guidance (99, 100). A recent unified framework for CCRs has been developed (101), and testing this unified approach with SUD treatment centers and other clinical settings is a future direction for research.

In sum, to address barriers to successful IPV screening and referral programs, best practices recommend that SUD and other clinical settings provide continuous and culturally-appropriate training to staff and providers about the prevalence of IPV, the co-occurrence of IPV with substance use, mental health, and physical health conditions, and protocols for screening and referring for IPV-related services (33, 37, 58, 92). Additionally, providing staff and providers with their own supports is needed to ensure their self-care and prevent burnout. Diversifying the workforce is one way to further address disparities in IPV screening and referral programs, and research from a range of healthcare fields shows that workforce diversity enhances patient-provider communication, increases healthcare satisfaction, and bolsters patient outcomes (102–104). Thus, it is crucial to hire people from diverse backgrounds and identities into a range of positions, and research supports that a diverse workforce is crucial from a patient-care perspective as well. Integrating more and developing novel technological tools may further improve access to IPV screening and referral and reduce disparities (30). Technology can standardize training and assessment across providers and clinics, increase equitable screening of patients through systematization (e.g., overcome potential provider biases or stereotypes), and be integrated into existing electronic medical records as part of standard workflows. Finally, increasing resources for and funding mechanisms to improve direct connection to IPV services is greatly needed. Additional development of and funding for IPV-support agencies that are culturally responsive and reach underserved populations are particularly needed. For example, more IPV-support agencies in rural areas, for LGBT+ individuals, and for minoritized individuals would help ensure that community needs are met. Assuring providers are trained and aware of resources is key as well.

### Inclusion of screening of co-occurring conditions

As noted above, few evidence-based IPV screening programs formally integrate additional assessment of co-occurring problems (e.g., substance use, depression, PTSD). However, IPV frequently co-occurs with substance use and other mental health conditions and here is some research to suggest that comprehensive screening for co-occurring conditions may be beneficial. For instance, trauma-informed screening, brief intervention, and referral to treatment (T-SBIRT) may be one way to improve mental health outcomes among individuals who screen positive for IPV (105). One study of adults (N = 83) receiving services from employment service programs showed that T-SBIRT, which screened for trauma exposure, PTSD, depression, anxiety, global mental health, physical health, and healthcare access, resulted in high rates of trauma-exposure detection and acceptance of referrals to mental health care (105). Another study using data collected from participants in the same employment services programs (N = 88) showed that those who completed the T-SBIRT reported reductions in depression and PTSD symptoms at follow-up relative to a comparison group who did not complete the T-SBIRT (106). This suggests that screening programs that address trauma exposure, such as IPV, and co-occurring conditions may directly affect the uptake of mental health referrals and be associated with improved distal mental health outcomes.

Future research could expand on this work by developing, testing, and utilizing integrated programs and comprehensive assessment toolkits that screen for IPV, substance use, and other co-occurring conditions. In the absence of more comprehensive and cohesive toolkits for screening IPV alongside commonly co-occurring conditions, existing SBIRTs can be integrated with IPV screeners in clinical practice to give providers a fuller scope of patients’ presenting concerns. While many SUD clinics may screen for mental health conditions on top of doing a thorough substance use assessment, it is critical that these settings also screen for IPV. Again, as stated above, individuals with SUD tend to not be screened for IPV, so ensuring that IPV assessment is part of standard screenings or an intake assessment battery is crucial for improving patient safety and overall health and well-being (29, 52, 78).

## Conclusions

While there is much research documenting the links between IPV and substance use, the current literature review identified that systematic, evidence-based IPV screening and referral programs are not routinely assessed in SUD treatment settings and there may be a need for comprehensive screeners that assess for the co-occurrence of both conditions as well as other psychiatric sequalae to best inform treatment and referral recommendations. Further, few screening programs include screening for IPV perpetration and there are significant disparities in who is screened and referred for IPV. Much work is needed to tackle these disparities and support universal IPV screening and resources for all people, regardless of their identities. IPV screening is generally effective at detecting IPV and seen as valuable by both patients and providers. Addressing current implementation barriers and limitations of existing IPV screening programs in SUD treatment settings is feasible, and education, training, advocacy, increased and streamlined resources, and technological tools will help us address these challenges in targeting and reducing substance-related IPV.

## Author contributions

AJ: Writing – original draft, Writing – review & editing. TS: Writing – original draft, Writing – review & editing.
